# Supplementary material for: Tamarix hispida NAC Transcription Factor ThNAC4 Confers Salt and Drought Stress Tolerance to Transgenic Tamarix and Arabidopsis
Source: Plants (Basel). 2022 Oct 8;11(19):2647. doi: 10.3390/plants11192647 (PMC9570625; doi:10.3390/plants11192647)
Supplement: Supplementary file 1 [file plants-11-02647-s001.zip › plants-1876153-supplementary.pdf]

Supplementary Materials

Table S1. Primers used for cloning of the *ThNAC4* gene.

Genes	Forward and reverse primers (5'–3')	
ThNAC4	ATGGAAAACATTCCTGGAT	TTAATAGTAACCCCAAAGGTC
M13	CGCCAGGGTTTTCCCAGTCACGAC	GAGCGGATAACAATTTCACACAGG

Table S2. Primers used in constructing recombinant plasmid pROKII-ThNAC4.

Genes	Forward and reverse primers (5'–3')	
pROKII-*ThNAC4*	CTCTAGAGGATCCCCGGGATGGA AAACATTCCTGGAT	TCGAGCTCGGTACCCGGGTTAATA GTAACCCCAAAGGTC
ThNAC4	ATGGAAAACATTCCTGGAT	TTAATAGTAACCCCAAAGGTC

Table S3. Primers used in constructing recombinant plasmid pFGC5941-ThNAC4.

Genes	Forward and reverse primers (5'–3')	
ThNAC4-Cis	ATAAGGAAGTTCATTTCATTTG	CAATCAAATGAAGAGCCAAT
ThNAC4-Anti	CTTACTTACACTTGCCTTGGAG	ATCTGAGCTACACATGCTCAG
pFGC5941-Cis	CATGATTTAAATCAACAGATGAG ATTTCAGC	TTGGCGCCATGCCAAATTAGGAG GAGG
pFGC5941-Anti	CCTTAATTAAATGCCAAATTAGG AGGAGG	CGCGGATCCGCAACAGATGAGAT TTCAGC

Table S4. Primers used in constructing recombinant plasmid pBI121-ThNAC4-GFP.

Genes	Forward and reverse primers (5'–3')	
pBI121-GFP	ATGGAAAACATTCCTGGAT	ATAGTAACCCCAAAGGTCTC
pBI121-ThNAC4-GFP	TCTAGACTGGTACCCGGGATGGA AAACATTCCTGGAT	CTAGTCAGTCGACCCGGGATAGT AACCCCAAAGGTCTC

Table S5. Gene-specific primers used in real-time PCR.

Genes	GenBank number	Forward and reverse primers (5'–3')	
		Tamarix hispida	
ThNAC4	JQ974958	CTACTGGGAAGGACAAAG	ATTAGACATGATGGTGGGG
β-actin	FJ618517	AAACAATGGCTGATGCTG	ACAATACCGTGCTCAATAGG
α-tubulin	FJ618518	CACCCACCGTTGTTCCAG	ACCGTCGTCATCTTCACC
β-tubulin	FJ618519	GGAAGCCATAGAAAGACC	CAACAAATGTGGGATGCT
		Arabidopsis thaliana	
α-tubulin	AT1G50010	GATGTACCGTGGTGATGTC	GAGCCTCTGAAAATTCTCC

Table S6. Primer sequences of SOD, POD and Trehalose synthase genes used in real-time PCR.

Genes	GenBank number	Forward and reverse primers (5'–3')	
α-tubulin	At1G50010	GATGTACCGTGGTGATGTC	GAGCCTCTGAAAATTCTCC
Ubiquitin	AT1G55060	GGAAAGCAGCTCGAAGATG	AAGCTTCCACCGCGGAGAC
SOD1	AT1G12520	GTCACCCGGAACCCACAGC	CCGAATAAAAGGCCTCTCC
SOD2	AT3G56350	GAAGGAGGTGGCAAACCAC	TCTTGTACTGTGGATAGTAG
SOD3	AT5G23310	CGCTGCACAGGTCTATAACC	AATATCGTCCCACACGAGTG
SOD4	AT5G51100	CCTGGAGGTGGAGGAAAGC	CTGCATTGGGCGTCTTCAC
POD1	AT1G05260	CTTTCACAAACCGTCTCTAC	AGTGGTGAGAGCAGAGTCTG
POD2	AT1G14550	CCATAGGACAATCTCAATGC	TGATCGGTTACTAATAGTC
POD3	AT1G24110	TCTGACCGTTCAAGAAATGG	TGGAGCAACCCGTAACCGTG
POD4	AT1G30870	TGTGGCACCATCCAGTCGAG	CTGCGAAAGTCTTTACAAGC
POD5	AT1G65970	CAGTATGAGCCATGTGCCTG	CAAGCAACAAAGCGAATCTC
POD6	AT2G18140	TCCGGGAGCCACACCATTGG	TGGTCGGAATTCAACAGTC
POD7	AT2G18150	CCAATCCGGAAACGGAAGTC	TCTGCATACTTCTTGACGAG
POD8	AT3G49110	GCAACACTGGATTACCTGAC	CCATCAGCATATGCTCTCAC
POD9	AT3G50990	AGGTTATACAACCATACTGG	CGTAATACTTGACCATCTC
POD10	AT4G11290	TCGACAGCGAATATGCCGAC	GAACTCTTGCTCCGATCCTC
POD11	AT4G17690	GAATGGTTTCACTCTAAAGG	GGAAGCTAACAGTCCAAGAC
POD12	AT4G25980	GAACAACGGCCTGCTTCTTC	TCCACGACCTGTCTGGTCG
POD13	AT4G26010	TCCAGGACAGGCTTTCCGAC	GAAGAGTGTATTGCTTGATG
POD14	AT4G30170	AGCCGTCACGGCCTCTCTC	CAAGATTTGATCTGACGTG
POD15	AT5G47000	GACTGTTCCTGACATCCAC	CTTGAAGTACATGTTGTCG
POD16	AT5G51890	CTTGTCGGTGAAAGACATG	GACCCAAACACTCCTTTCC
POD17	AT5G58390	ATCCCTCCTCCGATCACTAC	GTCGAACCTATCGGGAGAG
POD18	AT5G58400	GGCAAGCCAGGTGCGTCAC	TCCGGCTGTAGGATACGAC
POD19	AT5G66390	CTCACTAAGTTCAAGCGTC	GAATAGGGTCTGGTCACCTC
POD20	AT5G64110	CTGGACATACGATAGGAACG	GACTCGAGGAGACCTCGAC
TPS1	NM106505	TCCGACATGCCAGCCATTGC	TCTCTCCTTTGAGGTCAAGC
TPS7	NM001331627	CCAGATGGCTAAAGAAGAGG	GCAAACACATTTCCCTGATG
TPS8	NM001334443	GAAAGTAATCCGAGAAATGG	AGCACGTCGGCTTCATCGTC
TPS11	NM127426	TTAAACCTCAGGGAGTAAGC	ACACTTGGGGTATCATCGAG
TPPB	NM106458	ATGGGACAAGGGCCAGGCAC	ACTTGTTAACCTGAGAAGG
TPPC	NM102071	TATCCCTGGAGCTACGGTC	GCATCTTCATCAGTACGGTC
TPPD	NM103289	TCAAAAGGACTGGGGATTGG	CCTTGAAAGCATCCTCGTC
TPPF	NM117313	GTTTGCGTCTAACTCATGG	CTCGGTTCCCATCTCTCAG
TPPG	NM118385	TCTCGGATTAAGCAACAAC	CCCCCATTTCACCAAAGTC
TPPH	NM001342553	AAGTTTTGGAGGTTCGTCC	CCTCATCGGGTTCTTGCAG
TPPI	NM121048	GAATGGGATAAAGGAAAGG	TCTTGCAAAGAATACGAAGC
TPPJ	AK221501	GTTGAAACTGTCTCAAGGTC	AGCCTTGTCCTCTCCCTCG

Table S7. Primer sequences used in construction pGBKT7-ThNAC4 vector.

Genes	Forward and reverse primers (5'–3')	
pGBKT7-ThNAC4	CATGGAGGCCGAATTCATGGAAA ACATTCCTGG	GCAGGTCGACGGATCCTTAATAGT AACCCCAAAG
Rec2-1	CATGGAGGCCGAATTCATGGAAA ACATTCCTGGATT	GCAGGTCGACGGATCCCTGAAGA AATACCATTCC
Rec2-2	CATGGAGGCCGAATTCTTCAGTGT CAGAGACAGG	GCAGGTCGACGGATCCTCCTGCA CTCTTCTGAAAG
Rec2-3	CATGGAGGCCGAATTCGCAGGAT GTAAGAAGGTTC	GCAGGTCGACGGATCCCCATAGA ATGAGTTCAACGC
Rec2-4	CATGGAGGCCGAATTCATGGATC TGAATTCAATCCG	GCAGGTCGACGGATCCTTAATAGT AACCCCAAAGGTC
Rec2-5	CATGGAGGCCGAATTCATGGGAA TTGCGTGAACG	GCAGGTCGACGGATCCCCAACAA GTGGTTTTCC
Rec2-6	CATGGAGGCCGAATTCATGAATA TAGACTGGAGGG	GCAGGTCGACGGATCCGGTGAAG AGTCCGTCAATGG
Rec2-7	CATGGAGGCCGAATTCATGCCCG GTTATCATGCC	GCAGGTCGACGGATCCCGCCCTC CGTTCCGTTCC

Table S8. Primer sequences used in construction effector of pGADT7-rec2-ThNAC4.

Genes	Forward and reverse primers (5'–3')	
pGBKT7-ThNAC4	GCAGAGTGGCCATTATGGCCCAT GGAAAACATTCCTGG	GCGGCCGACATGTTTTTCCCTTA ATAGTAACCCCAAAG
pGADT7-Rec2	ATGAACATGGAGGCCAGTG	GATGGATCCCGTATCGATG

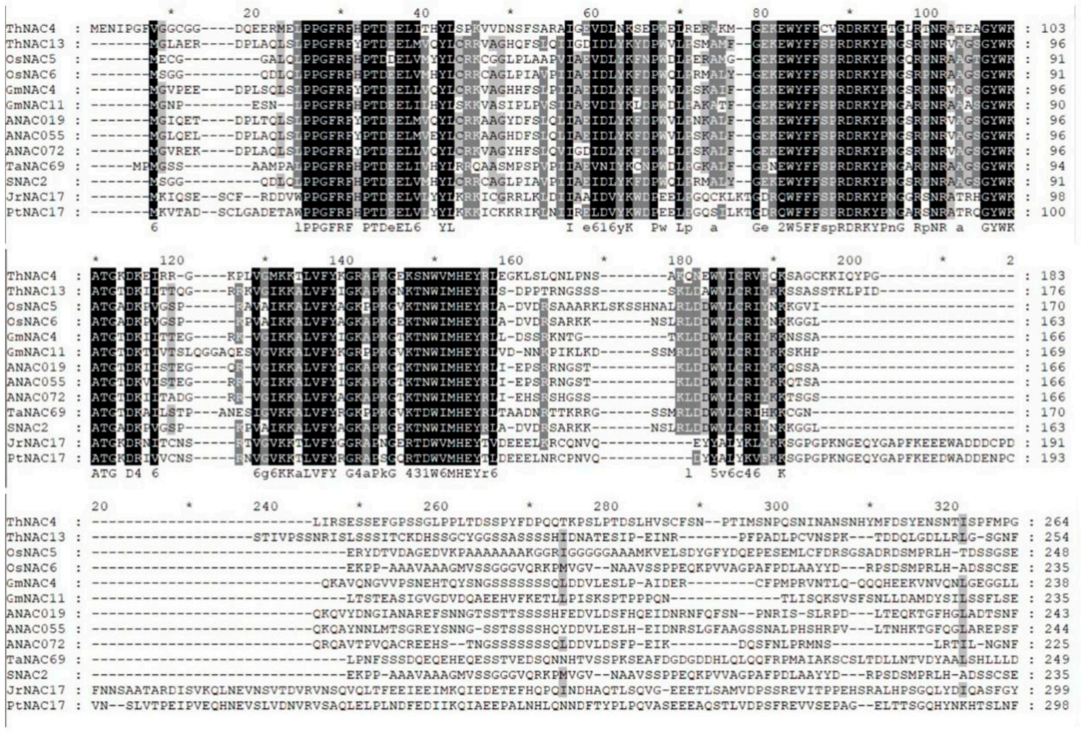

Fig. S1 Multiple sequence alignment analysis of NAC proteins from 12 other species

Multiple sequence alignments of ThNAC4 and 12 representative plant NACs were performed with ClustalX. The consensus NAC subdomains are shown in black color. Their corresponding accession numbers are as the follows: *Tamarix hispida* ThNAC4 (JQ974958) and ThNAC13 (JQ974967); *Oryza sativa* OsNAC5 (BAA89799), OsNAC6 (BAA89800) and SNAC2 (CBX55846); *Glycine max* GmNAC4 (AAY46124) and GmNAC11 (ACC66315); *Arabidopsis thaliana* ANAC019 (NP_175697.1), ANAC055 (NP_188169.1), and ANAC072 (NP_567773.1); *Triticum aestivum* TaNAC69 (AAU08785); *Juglans regia* JrNAC17 (XP_018848079.1); *Populus trichocarpa* PtNAC17 (AOF43232.1).

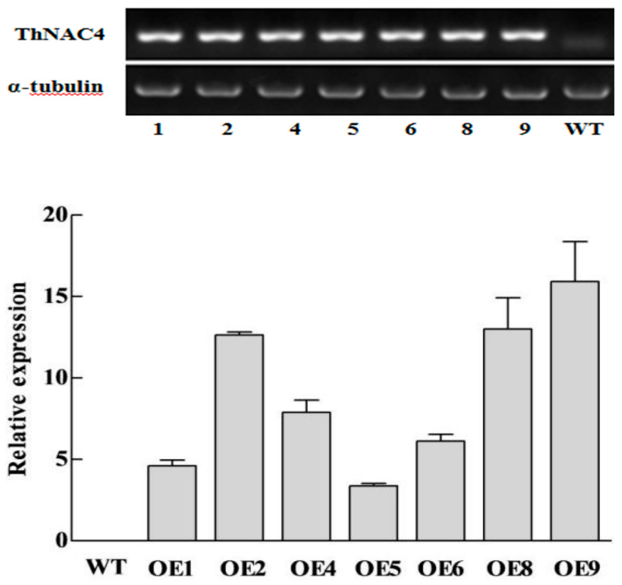

Fig. S2 Quantitative RT-PCR analysis of ThNAC4 expression in the WT and 9 homozygous overexpression lines (Line1-9) of ThNAC4-transformed *Arabidopsis*

Parallel reactions using *α-tubulin* (AT1G50010, as an internal control) in primers were carried out to normalize the amounts of added template.

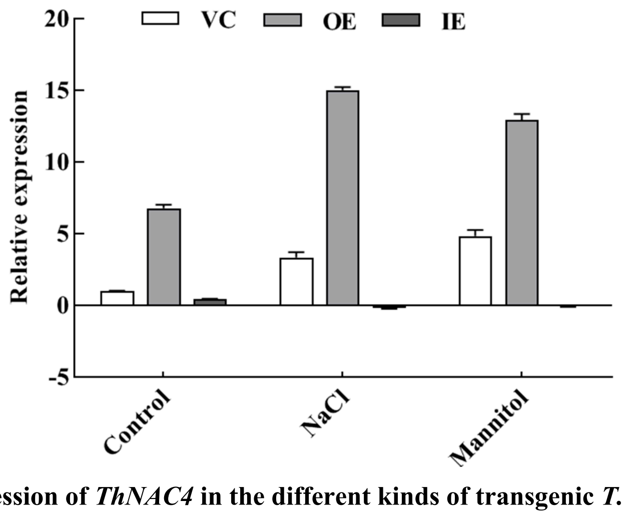

Fig. S3 Expression of *ThNAC4* in the different kinds of transgenic *T.hispida* plants

The expression of *ThNAC4* was determined under normal growth conditions or treatment with 150 mM NaCl or 200 mM mannitol for 24 h. The expression level of *ThNAC4* in control plants under normal growth conditions was used as the calibrator (designed as 1). VC: the pROKII vector control transformed *T. hispida* plants; OE: overexpressing of *ThNAC4* in *T. hispida* plants; IE: *ThNAC4* RNAi-silenced *T. hispida* plants. The error bars were standard deviations, which were calculated from multiple replicates of the real-time PCR.
